# Retraction Note: The role of lymph node dissection in the surgical treatment of endometrial cancer patients (retrospective analysis)

**DOI:** 10.1007/s00432-024-05690-w

**Published:** 2024-03-22

**Authors:** Oksana Movchan, Valentin Svintsitskyi

**Affiliations:** Research Department of Oncogynecology, National Cancer Institute, 33/43 Lomonosov Str, Kiev, 03022 Ukraine

**Retraction Note: Journal of Cancer Research and Clinical Oncology (2023) 149:63–68** 10.1007/s00432-022-04406-2

The Editor-in-Chief has retracted this article. An investigation by the Publisher has found a number of articles, including this one, which share similar concerns, involving but not limited to, irregularities with respect to submission and peer review. The authors have also been unable to provide documents confirming that ethics approval was obtained for the study. Additionally, there is a significant overlap with a previously-published article by the same authors (Movchan and Svintsitskyi 2022) that was not cited. The authors did not state explicitly whether they agree to this retraction.
